# Relapse recovery in relapsing–remitting multiple sclerosis: An analysis of the CombiRx dataset

**DOI:** 10.1177/13524585231202320

**Published:** 2023-10-13

**Authors:** Marcus W Koch, Ester Moral, Luis Brieva, Jop Mostert, Eva MM Strijbis, Jacynthe Comtois, Pavle Repovic, James D Bowen, Jerry S Wolinsky, Fred D Lublin, Gary Cutter

**Affiliations:** Departments of Clinical Neurosciences and Community Health Sciences, University of Calgary, Calgary, AB, Canada; Department of Neurology, Hospital Sant Joan Despí Moisès Broggi, Barcelona, Spain; Neuroimmunology Group, Department of Medicine, University of Lleida-IRBLleida, Lleida, Spain Department of Neurology, Hospital Universitari Arnau de Vilanova, Lleida, Spain; Department of Neurology, Rijnstate Hospital, Arnhem, The Netherlands; Department of Neurology, MS Center Amsterdam, Amsterdam University Medical Centers, Amsterdam, The Netherlands; Department of Medicine, Neurology Service, Maisonneuve-Rosemont Hospital, Montreal, QC, Canada; Multiple Sclerosis Center, Swedish Neuroscience Institute, Seattle, WA, USA; Multiple Sclerosis Center, Swedish Neuroscience Institute, Seattle, WA, USA; Department of Neurology, McGovern Medical School, University of Texas Health Science Center at Houston (UTHealth), Houston, TX, USA; Department of Neurology, Icahn School of Medicine at Mount Sinai, New York, NY, USA; Department of Biostatistics, University of Alabama at Birmingham, Birmingham, AL, USA

**Keywords:** Relapsing–remitting, steroids, treatment response, multiple sclerosis, clinical trial

## Abstract

**Background:**

Clinical relapses are the defining feature of relapsing forms of multiple sclerosis (MS), but relatively little is known about the time course of relapse recovery.

**Objective:**

The aim of this study was to investigate the time course of and patient factors associated with the speed and success of relapse recovery in people with relapsing–remitting MS (RRMS).

**Methods:**

Using data from CombiRx, a large RRMS trial (clinicaltrials.gov identifier NCT00211887), we measured the time to recovery from the first on-trial relapse. We used Kaplan–Meier survival analyses and Cox regression models to investigate the association of patient factors with the time to unconfirmed and confirmed relapse recovery.

**Results:**

CombiRx included 1008 participants. We investigated 240 relapses. Median time to relapse recovery was 111 days. Most recovery events took place within 1 year of relapse onset: 202 of 240 (84%) individuals recovered during follow-up, 161 of 202 (80%) by 180 days, and 189 of 202 (94%) by 365 days. Relapse severity was the only factor associated with relapse recovery.

**Conclusion:**

Recovery from relapses takes place up to approximately 1 year after the event. Relapse severity, but no other patient factors, was associated with the speed of relapse recovery. Our findings inform clinical practice and trial design in RRMS.

## Introduction

Relapses are the defining clinical feature of relapsing forms of multiple sclerosis (MS). In MS, relapses correlate with newly forming demyelinating lesions in the brain and spinal cord. Most clinical trials in relapsing–remitting MS (RRMS) use the number or annualized rate of clinical relapses as their primary outcome measure. It has been known since the 1960s that corticosteroid treatment (initially with adrenocorticotropic hormone (ACTH))^1^ can hasten the recovery from a relapse, and the disease modifying treatments (DMTs) for RRMS introduced since the 1990s both reduce the number and severity of relapses. Despite these successes in the treatment and prevention of relapses, relatively little is known about the time course of the recovery from relapses, and which patient factors are associated with the speed and success of recovery.

Clinical trial datasets give us an opportunity to study the time course of relapse recovery. While relapses occur at random time points throughout a trial, trial participants who experience a relapse are usually evaluated at an unscheduled study visit close to the relapse event, and then continue their regular trial follow-up afterward. This means that there is a record of a pre-relapse, an at-relapse, and often several post-relapse study visits, which makes it possible to study the time course of relapse recovery longitudinally and investigate the factors associated with the time to relapse recovery. Furthermore, most RRMS patients today are treated with DMTs, so that participants in clinical trials using these DMTs are a good representation of the patient population seen in clinical practice.

In this study, we used patient-level data from CombiRx, a large phase 3 trial of people with RRMS treated with the DMTs glatiramer acetate (GA), interferon beta (IFNB), or both, to investigate relapse recovery in people with RRMS.

## Methods

### Standard protocol approvals, registrations, and patient approvals

The ethical approval for CombiRx is described in the original trial publication.^2^ Ethical approval for this analysis was sought and granted by the Conjoint Health Research Ethics Board at University of Calgary and the Institutional Review Board at the University of Alabama at Birmingham. All participants gave written informed consent to their participation in CombiRx.

### CombiRx dataset

CombiRx was a three-arm, randomized, double-blind, placebo-controlled, multicenter, phase 3 trial of GA plus placebo (25%), or IFNB plus placebo (25%), or the combination of GA and IFNB (50%) in treatment-naïve people with early RRMS. Trial participants were followed until the last trial participant reached 3 years of follow-up. For these analyses, we included all study visits up to the 42-month visit. The inclusion criteria were age 18–60 years inclusive, a diagnosis of RRMS by Poser et al.^3^ or 2001 McDonald et al.^4^ criteria, and an Expanded Disability Status Scale (EDSS)^5^ of 0–5.5 inclusive. Trial participants needed to have at least two relapses in the 3 years before inclusion, where one relapse could be a magnetic resonance imaging (MRI) change meeting the 2001 McDonald MRI criteria for dissemination in time.^4^ Exclusion criteria were any prior use of IFNB or GA, an acute exacerbation within 30 days of screening, steroid use for acute exacerbations within 30 days of screening, chronic systemic steroid use, evidence of progressive MS, and any previous treatment with natalizumab, cladribine, alemtuzumab, daclizumab, rituximab, or total lymphoid irradiation.

### Relapse recovery

In CombiRx, relapses were defined as new or worsening symptoms attributable to MS, preceded by 30 days of stability, lasting for more than 24 hours, not associated with fever, and leading to ⩾ 0.5 EDSS points increase compared to a prior visit or ⩾ 2 points increase in one EDSS functional system, or ⩾ 1 point increase in two EDSS functional systems (excepting bladder and cognitive changes) as assessed by a treatment-blinded observer. Relapses were defined as a “protocol-defined exacerbation” if an EDSS assessment took place within 7 days after relapse onset, and as a non–protocol-defined exacerbation if the EDSS assessment occurred more than 7 days after relapse onset.^2^ For this study, we combined these two categories into a single “confirmed relapse” category which is consistent with relapse definitions in most clinical trials.

For our analyses, we selected the trial participants’ first confirmed relapse. Included relapses had to have the at-relapse EDSS assessment within 30 days from relapse onset, a relapse severity (the difference between the at-relapse and pre-relapse EDSS) of at least 0.5 points, and at least one post-relapse EDSS assessment. We marked relapse recovery at the first instance; a post-relapse EDSS was equal or smaller than the pre-relapse EDSS. Trial participants were censored at the time of their last EDSS assessment, or at the time of a second confirmed relapse. In addition to unconfirmed relapse recovery, we investigated 12- and 24-week confirmed relapse recovery. For the two confirmation cohorts, we selected trial participants who had at least one additional EDSS assessment at least 12 or 24 weeks after the recovery event.

### Additional analysis: illustration of short-term EDSS fluctuation

To illustrate the occurrence of short-term fluctuation in EDSS measurements in the absence of relapses, especially in its lower ranges, we compared screening and baseline EDSS measurements. We first selected all CombiRx participants with a screening EDSS score of 0.0–3.0. In CombiRx, the screening and baseline visits occurred at most 45 days apart and participants had to be relapse-free within 30 days of the screening visit. We then compared screening and baseline EDSS, and recorded the percentage of participants with identical screening and baseline scores, and the proportion of participants with higher and lower baseline than screening EDSS scores.

### Statistical analyses

We used Kaplan–Meier survival analyses and Cox regression models to investigate the association of the factors sex, age at baseline, disease duration at baseline, treatment arm, pre-relapse EDSS (in the categories “0.0,” “1.0–2.0,” and “> 2.0”), number of relapses in the year before inclusion, contrast-enhancing lesions (CELs) on the baseline MRI scan (yes/no), burden of disease (BOD, in mL) on the baseline MRI scan, high-dose steroid treatment of the relapse (yes/no), and relapse severity (in the categories “0.5 EDSS points,” “1.0 EDSS points,” and “> 1.0 EDSS points”) with the time to relapse recovery. To investigate the possible interaction between relapse severity and high-dose steroid treatment, we included an interaction term for these variables in all models. We used the *R* statistical software package for Windows, version 4.2.2 for all statistical analyses.^6^ Statistical significance was assumed to be at the two-sided 0.05 level.

### Data availability

Access to the CombiRx dataset can be requested from the Coordinating Center or MS Center at the University of Alabama at Birmingham (Birmingham, Alabama, USA) by completing a data use agreement that is reviewed by a committee overseeing the use of the data. Qualified researchers have or will obtain appropriate Institutional Review Board approval for the study request. Depending on the complexity of the request, researchers may need to cover the cost of producing the de-identified data.

## Results

### CombiRx dataset

The CombiRx dataset contained individual patient-level data of 1008 participants. Table 1 shows their baseline characteristics. The treatment arms were well balanced with a slightly older average age for GA (Table 1).

**Table 1.** Baseline characteristics of the CombiRx participants.

	GA	IFNB	GA and IFNB	All
*n*	259	250	499	1008
Female, *n* (%)	185 (71)	173 (69)	372 (75)	730 (72)
Mean age, years (*SD*)	39.0 (9.5)	37.6 (10.2)	37.1 (9.4)	37.7 (9.7)
Mean disease duration, years (*SD*)	1.0 (2.9)	1.4 (4.0)	1.1 (3.1)	1.2 (3.3)
Mean relapses in the year before the study (*SD*)	1.6 (0.7)	1.7 (0.9)	1.7 (0.7)	1.7 (0.8)
Median EDSS (*IQR*)	2.0 (1.0–2.5)	2.0 (1.0–2.5)	2.0 (1.0–2.5)	2.0 (1.0–2.5)
Individuals with CELs, *n* (%)	106 (41)	104 (42)	189 (38)	399 (40)
Mean BOD, mL (*SD*)	12.9 (13.7)	11.7 (12.2)	12.1 (13.5)	12.2 (13.2)

GA: glatiramer acetate; IFNB: interferon beta; GA and IFNB: combination of glatiramer acetate and interferon beta; SD: standard deviation; IQR: interquartile range; CELs: contrast-enhancing lesions, BOD: burden of disease.

### Relapse recovery

Table 2 shows the characteristics of the relapses included in the analysis on unconfirmed and 12- and 24-week confirmed relapse recovery. We identified 240 relapses matching the inclusion criteria. As expected, the confirmed cohorts included considerably fewer participants, 167 (69.6%) in the 12-week confirmed and 156 (65.0%) in the 24-week confirmed cohort. The pre-relapse EDSS was very similar to the baseline EDSS, both with a median of 2.0 (interquartile range, IQR, of 1.5–2.5) in all cohorts. The median times between the pre-relapse EDSS and relapse onset, the at-relapse EDSS, and relapse severity were similar in the three cohorts (Table 2). The median time between relapse onset and the at-relapse assessment was 6 days (IQR 4–13) in the unconfirmed, 7 days (IQR 4–12) in the 12-week confirmed, and 6.5 days (4–12) in the 24-week confirmed cohort.

**Table 2.** Characteristics of the analyzed relapses.

	Unconfirmed recovery	12-week confirmed recovery	24-week confirmed recovery
No. of participants with any evaluable relapse (*n*)	240	167	156
Female, *n* (%)	175 (73)	122 (73)	113 (72)
Age at baseline, years (median, IQR)	36 (28–44)	36 (29–44)	36 (29–44)
EDSS at baseline (median, IQR)	2.0 (1.5–2.5)	2.0 (1.5–2.5)	2.0 (1.5–2.5)
Pre-relapse EDSS (median, IQR)	2.0 (1.5–2.5)	2.0 (1.5–2.5)	2.0 (1.5–2.5)
Days between pre-relapse assessment and relapse onset (median, IQR)	46 (22–62.25)	45 (22–65)	44.5 (21–65.5)
At-relapse EDSS (median, IQR)	3.0 (2.0–3.5)	3.0 (2.5–3.5)	3.0 (2.5–3.5)
Relapse severity ΔEDSS (median, IQR)	1.0 (0.5–1.5)	1.0 (0.5–1.5)	1.0 (0.5–1.5)
Relapse severity categories, *n* (%)			
ΔEDSS 0.5 EDSS points	74 (31)	57 (34)	52 (33)
ΔEDSS 1.0 EDSS points	75 (31)	56 (34)	55 (35)
ΔEDSS > 1.0 EDSS points	91 (38)	54 (32)	49 (31)
Days relapse onset and at-relapse assessment (median, IQR)	6 (4–13)	7 (4–12)	6.5 (4–12)
Participants receiving high-dose steroid treatment, *n* (%)	129 (54)	86 (52)	82 (53)
Participants with recovery, *n* (%)	202 (84)	86 (52)	85 (55)
Recovery (*n* (%) of recovered participants):			
Within 30 days	10 (4)	5 (12)	7 (8)
Within 90 days	91 (45)	48 (56)	49 (58)
Within 180 days	161 (80)	77 (90)	73 (91)
Within 365 days	189 (94)	85 (99)	84 (99)
Later than 365 days	13 (6)	1 (1)	1 (1)

IQR: interquartile range.

**Figure 1.**
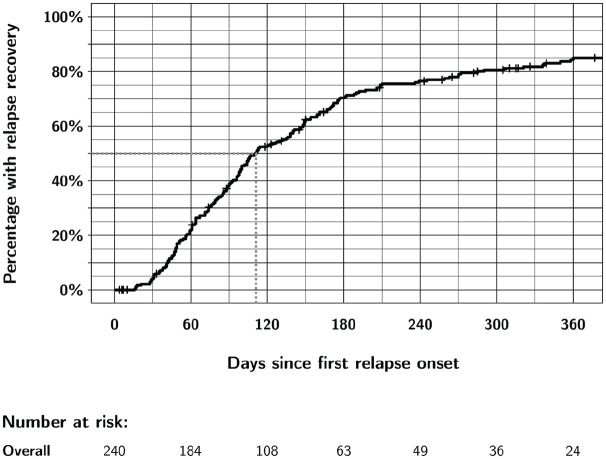
Overall unconfirmed recovery from first relapse in CombiRx participants up to 1 year after relapse onset. Relapse recovery in CombiRx appears to occur in two phases. There is a steady almost linear increase in recovery events up to about 6 months, with fewer additional recovery events afterward. More than 80% of relapses had recovered at the 1-year mark. Participants were censored at the time of their last EDSS assessment, or at the time of a second confirmed relapse. The gray dotted line represents median time to relapse recovery.

Table 2 and Figure 1 show the time course of relapse recovery. The median time to relapse recovery was 111 days (95% CI: 99–138). Most recovery events took place within 1 year of relapse onset: for example, in the unconfirmed relapse recovery cohort, 202 of 240 (84%) individuals recovered during follow-up and 189 of the 202 (94%) during the first 365 days after relapse onset (Table 2). Relapse recovery was not linear over time: 80% of those who recovered did so within the first 6 months (Table 2 and Figure 1).

In addition to unconfirmed relapse recovery, we investigated 12- and 24-week confirmed relapse recovery (Table 2). Since these cohorts required additional follow-up time points, the confirmed cohorts included fewer individuals. When 12- and 24-week confirmation was mandated, there were much fewer recovery events: only 52% of the 12-week confirmed cohort and only 55% of the 24-week confirmed cohort experienced relapse recovery, compared to 84% in the unconfirmed cohort. However, the time course of recovery was roughly similar in the unconfirmed and confirmed cohorts (Table 2).

### Factors associated with relapse recovery

Table 3 shows the results of the Cox regression model for the unconfirmed cohort. We deem unconfirmed relapse recovery to be the most clinically relevant, since most clinicians would likely accept that a patient has recovered from a relapse if their EDSS score reached the pre-relapse level. Furthermore, since the unconfirmed cohort was far larger than the confirmed cohorts, it is the most attractive to analyze in the interest of the precision of the estimated hazard ratios in the Cox regression models. Only relapse severity was significantly associated with relapse recovery in our cohort: participants with a relapse severity of more than 1.0 EDSS point were significantly less likely to experience relapse recovery with a hazard ratio for relapse recovery of 0.58 (95% CI: 0.34–0.98) compared to participants with 0.5 EDSS points relapse severity (Table 3). All other investigated factors, which included clinical, treatment, and MRI characteristics, were not associated with relapse recovery.

**Table 3.** Cox regression model of factors associated with the time to unconfirmed relapse recovery.

Risk factor	Hazard ratio (95% CI)	*p*
Sex		
Female	1.00 (reference)	—
Male	0.89 (0.62–1.26)	0.50
Age at baseline^a^	1.00 (0.98–1.01)	0.70
Disease duration^a^	0.98 (0.95–1.01)	0.22
Treatment arm		
GA	1.00 (reference)	—
IFNB	1.22 (0.80–1.87)	0.22
GA and IFNB	1.28 (0.87–1.88)	0.20
Pre-relapse EDSS		
0.0	1.00 (reference)	—
1.0–2.0	1.09 (0.70–1.7)	0.22
>2.0	1.25 (0.76–2.07)	0.25
Relapses in the year before inclusion^b^	1.13 (0.96–1.33)	0.08
CELs at baseline		
No (reference)	1.00 (reference)	—
Yes	0.90 (0.66–1.22)	0.50
BOD at baseline^b^	1.00 (0.99–1.01)	0.73
Steroid treatment		
No (reference)	1.00 (reference)	—
Yes	1.43 (0.87–2.36)	0.16
Relapse severity		
0.5 EDSS points	1.00 (reference)	—
1.0 EDSS points	0.82 (0.48–1.39)	0.46
>1.0 EDSS points	0.58 (0.34–0.98)	0.04
Interaction term		
Steroid treatment: yes × Relapse severity 0.5 EDSS points	1.00 (reference)	—
Steroid treatment: yes × Relapse severity 1.0 EDSS points	0.82 (0.40–1.69)	0.60
Steroid treatment: yes × Relapse severity > 1.0 EDSS points	0.81 (0.40–1.640)	0.56

a Per year increase.

b Per unit increase.

**Figure 2.**
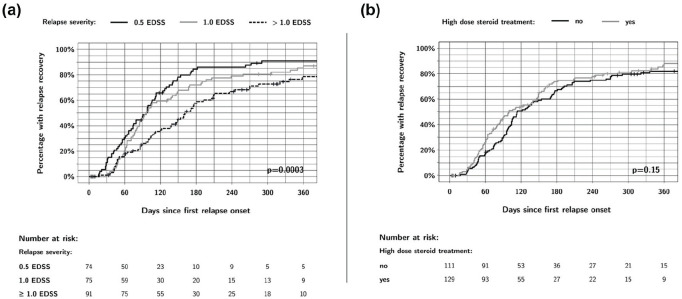
Effect of relapse severity and high-dose steroid treatment on relapse recovery up to 1 year of follow-up. (a) A relapse severity of more than 1.0 EDSS point was significantly associated with slower relapse recovery. (b) People receiving high-dose steroid treatment had a faster relapse recovery, especially in the first 4 months post-relapse, but this difference was not statistically significant.

The Kaplan–Meier curves for the risk factors relapse severity and high-dose steroid treatment are shown in Figure 2. It appears that participants receiving a high-dose steroid course have a faster recovery in the first few months, with the Kaplan–Meier curves separating up to approximately Month 4. However, this difference disappears afterward and does not reach statistical significance in either the Kaplan–Meier analysis (log-rank *p* = 0.15, Figure 2) or in the multivariable Cox regression model (hazard ratio 1.43, 95% CI: 0.87–2.36, *p* = 0.16, Table 3).

Tables 4 and 5 show the results of the Cox regression models for the 12- and 24-week confirmed cohorts, which showed largely similar results. In the 24-week confirmed cohort, relapse severity did not reach statistical significance, while having a pre-relapse EDSS of between 1.0 and 2.0 was associated with a greater chance for relapse recovery (Tables 4 and 5). The interaction term between relapse severity and high-dose steroid treatment was not significant in any of the models.

**Table 4.** Cox regression model of factors associated with the time to 12-week confirmed relapse recovery.

Risk factor	Hazard ratio (95% CI)	*p*
Sex		
Female	1.00 (reference)	—
Male	0.67 (0.37–1.21)	0.18
Age at baseline^a^	1.00 (0.89–1.03)	0.92
Disease duration^a^	0.96 (0.91–1.02)	0.22
Treatment arm		
GA	1.00 (reference)	—
IFNB	0.97 (0.51–1.84)	0.92
GA and IFNB	0.99 (0.55–1.78)	0.98
Pre-relapse EDSS		
0.0	1.00 (reference)	—
1.0–2.0	1.35 (0.66–2.75)	0.41
>2.0	0.92 (0.41–2.09)	0.84
Relapses in the year before inclusion^b^	1.18 (0.93–1.51)	0.08
CELs at baseline		
No (reference)	1.00 (reference)	—
Yes	0.88 (0.53–1.47)	0.64
BOD at baseline^b^	1.00 (0.99–1.02)	0.70
Steroid treatment		
No (reference)	1.00 (reference)	—
Yes	2.09 (0.99–4.37)	0.05
Relapse severity		
0.5 EDSS points	1.00 (reference)	—
1.0 EDSS points	1.02 (0.47–2.21)	0.97
>1.0 EDSS points	0.30 (0.11–0.81)	0.02
Interaction term		
Steroid treatment: yes × Relapse severity 0.5 EDSS points	1.00 (reference)	—
Steroid treatment: yes × Relapse severity 1.0 EDSS points	0.53 (0.18–1.56)	0.25
Steroid treatment: yes × Relapse severity > 1.0 EDSS points	1.27 (0.37–4.48)	0.70

a Per year increase.

b Per unit increase.

**Table 5.** Cox regression model of factors associated with the time to 24-week confirmed relapse recovery.

Risk factor	Hazard ratio (95% CI)	*p*
Sex		
Female	1.00 (reference)	—
Male	0.90 (0.51–1.59)	0.72
Age at baseline^a^	0.99 (0.97–1.01)	0.41
Disease duration^a^	0.99 (0.92–1.07)	0.88
Treatment arm		
GA	1.00 (reference)	—
IFNB	0.84 (0.43–1.64)	0.61
GA and IFNB	1.00 (0.55–1.79)	0.99
Pre-relapse EDSS		
0.0	1.00 (reference)	—
1.0–2.0	2.55 (1.15–5.64)	0.02
>2.0	1.20 (0.48–2.98)	0.69
Relapses in the year before inclusion^b^	1.10 (0.86–1.41)	0.46
CELs at baseline		
No (reference)	1.00 (reference)	—
Yes	1.30 (0.78–2.17)	0.31
BOD at baseline^b^	0.99 (0.97–1.01)	0.31
Steroid treatment		
No (reference)	1.00 (reference)	—
Yes	1.98 (0.90–4.35)	0.09
Relapse severity		
0.5 EDSS points	1.00 (reference)	—
1.0 EDSS points	0.77 (0.33–1.82)	0.55
>1.0 EDSS points	0.55 (0.22–1.36)	0.20
Interaction term		
Steroid treatment: yes × Relapse severity 0.5 EDSS points	1.00 (reference)	—
Steroid treatment: yes × Relapse severity 1.0 EDSS points	0.84 (0.27–2.64)	0.77
Steroid treatment: yes × Relapse severity > 1.0 EDSS points	0.98 (0.30–3.28)	0.98

a Per year increase.

b Per unit increase.

### Additional analysis: illustration of short-term EDSS fluctuation

In CombiRx, 849 participants had a screening EDSS score between 0 and 3.0. The median number of days between the screening and the baseline visit was 22 days (IQR 3–28 days). Of these 849 participants, 354 (41.7%) had identical screening and baseline scores, 231 (27.2%) had a higher baseline EDSS score compared to screening, and 264 (31.1%) had a lower baseline score compared to screening.

## Discussion

Despite the importance of clinical relapses in relapsing forms of MS, relatively little is known about the time course of relapse recovery. This is likely in part since people with MS in typical clinical practice are not seen often enough to have a pre-relapse assessment that is close to the relapse event, and often not followed up as closely as in a clinical trial afterwards. Clinical trial datasets are a valuable data source to address this question because participants are generally assessed every 3 months, assuring that a randomly occurring relapse during a trial is never farther removed from a scheduled assessment than these 3 months. Previous studies on relapse recovery were more focused on the residual disability from a relapse rather than on the time course of recovery. One such investigation used data from the placebo arms of several clinical trials in RRMS to determine the percentage of patients with and the magnitude of residual deficits following a relapse.^7^ This study had restricted access to trial data and focused on analyzing the difference between pre-, at-, and post-relapse EDSS scores after a varying time of follow-up: 224 people with RRMS were analyzed, and 42% had not fully recovered after an average of 64 days.^7^ This study is difficult to compare to our investigation because of differences in the data source, analyses, and purpose; but based on our analyses, it appears that further recovery occurs after longer follow-up, in our cohort for up to a year. Another study compared relapse recovery between placebo- and natalizumab-treated participants in the AFFIRM study.^8^ This investigation included 283 participants and found a substantial advantage in 12-week confirmed relapse recovery for natalizumab-treated patients. While this study is also difficult to compare to our investigation, it does contain survival curves showing a similar time course with the majority of recovery occurring in the first 6 months of follow-up, noticeably fewer recovery events between 6 and 12 months, and even fewer afterwards.^8^ The observed time course of relapse recovery raises the question of the appropriateness of the confirmation of disability worsening in RRMS trials. Clinical trials in RRMS often mandate that a disability worsening event be confirmed at a further assessment after 3 or 6 months. This practice is done in part with the intention of measuring “fixed” disability worsening, and to minimize the effect of relapses. However, our analyses suggest that relapses still influence these measures after 3 and 6 months, as 65% of people in the unconfirmed cohort recovered after the 3-month mark and 20% after the 6-month mark (Table 2). This would imply that some of the 3- or 6-month confirmed progression events in RRMS trials may in fact be recovering relapses.

Our analyses showed a marked difference in unconfirmed and confirmed relapse recovery: while 84% of trial participants experienced recovery to their previous baseline in the unconfirmed cohort, this percentage shrank to only 52% and 55% for the 12- and 24-week confirmed cohorts. We believe that two main factors are responsible for this difference. One important factor is trial participants who may not have had sufficient follow-up data to confirm the recovery: for example, almost 10% of subjects in the unconfirmed cohort (21 of 240) either completed the trial or had a relapse within 12 weeks of the recovery index date, and thus were not evaluable for confirmation. Another factor contributing to this difference is short-term variability that characterizes the EDSS, especially in its lower ranges. Koch et al.^9^ and Liu and Blumhardt^10^ have previously commented on this characteristic of the EDSS, which—for example—is also in part responsible for the substantial difference between confirmed and sustained disability progression if measured with the EDSS. To illustrate this point, we added a description of the short-term score change between the screening and the baseline EDSS measurements in CombiRx in the absence of relapses. In 58.3% of the 849 participants with a screening EDSS of 3.0 or lower, there was a difference of at least 0.5 EDSS points between the screening and baseline EDSS scores, with the baseline score higher than the screening score in 27.2% of participants. If a similar amount of random variation of the EDSS is present throughout the trial, 27.2%, or close to a third, of participants would not be confirmed due to measurement error, which is non-trivial. We observed 12-week confirmation in 52% of participants, thus 48% were not confirmed. Given the substantial short-term variation in EDSS, 27.2% of these 48% unconfirmed relapse recoveries might simply be due to measurement error. This would move the 52% confirmation percentage to 65.1% (52% + 0.272 × 48%) which suggests that the striking difference between unconfirmed and confirmed relapse recovery is certainly much less different than it appears. Given these considerations, it may be unnecessary to mandate confirmation for relapse recovery. While it is understandable that disability worsening, especially if it is used as the primary outcome measure in a trial, should be confirmed, most clinicians would likely agree that a patient has recovered from a relapse once they reach their pre-relapse disability level.

In our investigation of factors associated with relapse recovery, we found that greater relapse severity was associated with a lower chance of recovery. While this finding is expected, it is also relevant that many other factors were not associated with relapse recovery. We included factors associated with disease activity (relapse activity in the year before inclusion and MRI CELs at baseline), demographics (sex, age, disease duration), indicators of disease burden (pre-relapse EDSS and MRI BOD), and treatment (DMT treatment arm and high-dose steroid treatment) into our regression model; none impacted relapse recovery. As high-dose steroid treatment is very widely used in clinical practice, it may appear counter-intuitive that steroid treatment did not significantly affect relapse recovery. However, this finding is in keeping with the Optic Neuritis Treatment Trial, which showed that high-dose intravenous steroid treatment (with 1 g intravenous methylprednisolone for 3 days) hastened the recovery of visual acuity, but there was no statistically significant difference in visual acuity between high-dose steroid and placebo arms at 6 months of follow-up.^11^ Similarly, one could have expected that at least age may have an effect on the success of relapse recovery, as an observational study in 132 pediatric and 632 adult patients with MS found that children recover significantly better from relapses than adults.^12^ However, as noted above, there is high variability in the assessment of the EDSS and “false recovery” due to measurement error of the EDSS could also be a reason for the lack of statistical significance on some expected risk factors for recovery. Still, while one-third of the relapses were severe and were half as likely to recover even on first-generation DMT therapy, the linkage between disability worsening and relapses is evident.

Our study has several limitations. Since CombiRx was a trial including treatment-naïve patients with early MS with a mean disease duration of only 1.2 years at baseline, it is unclear whether our findings can be generalized to the entire spectrum of people with relapsing or progressive forms of MS who experience relapses. The longitudinal investigation of relapse recovery should be investigated in other clinical trial and real-world adult and pediatric MS datasets. Even though 1008 people participated in CombiRx, our cohort of patients with a first on-trial relapse included only 240 patients over an average follow-up period of over 3 years, this may have influenced the precision of the estimates.

In this study, we described the time course of and investigated factors associated with relapse recovery. Further studies into the effect of newer DMTs on relapse recovery, and on patient factors and biomarkers associated with relapse recovery are warranted.
